# Zerone: a ChIP-seq discretizer for multiple replicates with built-in quality control

**DOI:** 10.1093/bioinformatics/btw336

**Published:** 2016-06-10

**Authors:** Pol Cuscó, Guillaume J. Filion

**Affiliations:** ^1^Genome Architecture, Gene Regulation, Stem Cells and Cancer Programme, Centre for Genomic Regulation (CRG), the Barcelona Institute of Science and Technology, Barcelona 08003, Spain; ^2^Universitat Pompeu Fabra (UPF), Barcelona, Spain

## Abstract

**Motivation:** Chromatin immunoprecipitation followed by high-throughput sequencing (ChIP-seq) is the standard method to investigate chromatin protein composition. As the number of community-available ChIP-seq profiles increases, it becomes more common to use data from different sources, which makes joint analysis challenging. Issues such as lack of reproducibility, heterogeneous quality and conflicts between replicates become evident when comparing datasets, especially when they are produced by different laboratories.

**Results**: Here, we present Zerone, a ChIP-seq discretizer with built-in quality control. Zerone is powered by a Hidden Markov Model with zero-inflated negative multinomial emissions, which allows it to merge several replicates into a single discretized profile. To identify low quality or irreproducible data, we trained a Support Vector Machine and integrated it as part of the discretization process. The result is a classifier reaching 95% accuracy in detecting low quality profiles. We also introduce a graphical representation to compare discretization quality and we show that Zerone achieves outstanding accuracy. Finally, on current hardware, Zerone discretizes a ChIP-seq experiment on mammalian genomes in about 5 min using less than 700 MB of memory.

**Availability and Implementation**: Zerone is available as a command line tool and as an R package. The C source code and R scripts can be downloaded from https://github.com/nanakiksc/zerone. The information to reproduce the benchmark and the figures is stored in a public Docker image that can be downloaded from https://hub.docker.com/r/nanakiksc/zerone/.

**Contact**: guillaume.filion@gmail.com

**Supplementary information**: Supplementary data are available at *Bioinformatics* online.

## 1 Introduction

One of the major challenges of biology is to understand how transcription factors and chromatin proteins coordinate transcription, replication and repair. In front of this colossal task, the community invests massive research efforts into collecting protein-genome interaction data. Chromatin immunoprecipitation followed by high throughput sequencing (ChIP-seq) has become the standard method to identify the targets of a transcription factor or a histone modification in a cell population. However, ChIP is not fully understood and artifacts are still discovered more than 10 years after its adoption as a standard (Park *et al.*, 2013; Teytelman *et al.*, 2013). Besides, the constant improvement of sequencing technologies makes analysis of ChIP-seq profiles difficult to standardize. There is thus a need to continuously develop and improve computational tools to analyze ChIP-seq data.

One of the most common analyses performed on ChIP-seq profiles is to discretize the signal, i.e. identify the loci where the transcription factor (or other feature) is present. This makes the signal simpler to interpret, it removes part of the experimental noise, it simplifies downstream analyses and it allows comparing or combining profiles of different natures. This raises a challenge at the computational level because discretization has to be carried out uniformly for signals that may be very different. For instance, lamins bind in megabase-scale domains covering 40% of the genome (Guelen *et al.*, 2008), whereas transcription factors may bind as few as 6 bp with a coverage below 1%.

Large consortia such as ENCODE have brought to light severe issues related to the quality of ChIP-seq data. Conflicts between replicates are common, and sometimes laboratory effects are clearly detectable in the data, even when experimentalists use the same material and follow the same protocol (our unpublished observations). The most popular remedy is to use a metric called IDR (Irreproducible Discovery Rate, Li *et al.*, 2011), which allows weeding out poorly reproducible signal. This approach is a significant step forward, but the IDR is undefined when more than two replicates are available. Besides, keeping only the reproducible ChIP-seq peaks is not always the best option. If one of the replicates is mislabelled, for instance, it is more appropriate to reject the dataset than to keep the common peaks. In summary, how to integrate ChIP-seq data from different sources and with variable qualities is still an open problem.

Here, we propose an approach to discretize ChIP-seq data where conflict resolution and quality control are integrated in a tool that we called Zerone (Pronounced /ziˈroʊn/ or /ˈzi:ron/, i.e. as inserting ‘ear’ in ‘zone’.). The key idea of Zerone is to combine an arbitrary number of ChIP-seq replicates in a single discretized profile, where conflicts are resolved by maximizing the likelihood of the underlying statistical model. Following discretization, Zerone controls the quality of its output in order to detect potential anomalies, and when applicable rejects the output as a whole. Internally, the first step implements a Hidden Markov Model (HMM) with zero-inflated negative multinomial (ZINM) emissions, and the second implements a Support Vector Machine (SVM) trained using ENCODE ChIP-seq data. HMM-based discretization is agnostic about the shape of the signal (broad domains or sharp peaks) and the ZINM distribution captures the essential features of the read count distribution in ChIP-seq data.

Zerone is designed for large volume pipelines aiming to combine many ChIP-seq profiles with little human intervention. To this end, it is compatible with the standard BED, SAM/BAM and GEM formats, it produces congruent window-based outputs, and it can process hundreds of experiments per day on average hardware. We benchmarked Zerone against MACS (Zhang *et al.*, 2008), BayesPeak (Spyrou *et al.*, 2009) and JAMM (Ibrahim *et al.*, 2015) on the core task of discretizing ChIP-seq profiles of CTCF and H3K36me3. Our results show that Zerone is competitive in terms of speed and accuracy.

## 2 Methods

### 2.1 Model and parameter estimation

**Fig. 1.**
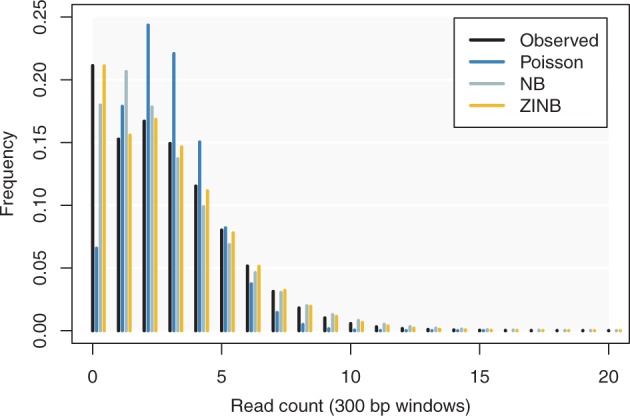
Using the ZINB distribution to model ChIP-seq data (color version of the figure available online). Reads from a mock control dataset were mapped onto the human genome and pooled in 300 bp windows after removing duplicates. The histogram of the read counts is shown in black (no immunoprecipitation was performed in this experiment, so this variation corresponds to the ‘baseline’). The colored histograms show the maximum likelihood fit of the Poisson, Negative Binomial (NB) and Zero-Inflated Negative Binomial (ZINB) distributions. The fit of the Poisson distribution is poor. The NB distribution gives a good fit at the tail, but not for windows with 0 and 1 read. The ZINB distribution gives a good fit over the whole range. Data from ENCODE file ENCFF000VEK (Color version of this figure is available at *Bioinformatics* online.)

It is natural to model read counts per genomic window by an unbounded discrete distribution. The Poisson distribution is an obvious candidate, but it is a poor choice because the variance of read counts is usually higher than the mean for ChIP-seq data. The reason is that windows are nonhomogeneous, which increases the dispersion. More specifically, windows do not have the same copy numbers, they are not equally PCR-prone and they are not equally mappable. The negative binomial (NB) distribution is thus a better choice because it allows some variation between windows. However, genomes are fraught with repeats, which creates an excess of windows where reads cannot be mapped. Since such windows will always have 0 read count, a natural choice for this distribution is the zero-inflated negative binomial (ZINB), i.e. the mixture of a negative binomial distribution and a distribution concentrated at 0 (Rashid *et al.*, 2011). Figure 1 shows that the ZINB distribution gives a better fit to ChIP-seq data than Poisson and NB distributions.

The ZINB distribution has three parameters that can be fitted by maximum likelihood. Zerone uses a custom solver based on the Newton-Raphson method, which converges much faster than the popular routine zeroinfl (Zeileis *et al.*, 2008) from the R package pscl (Jackman, 2015).

The NB distribution can be interpreted as a Gamma-Poisson process, which gives a straightforward extension to a multivariate distribution called the Negative Multinomial (NM) and to the zero-inflated version of it called Zero-Inflated Negative Multinomial distribution (ZINM, see supplementary material for detail). In this model, windows have an intrinsic ChIP-seq bias due to their sequence composition, mappability and other inherent properties, which gives a baseline variation present in all ChIP-seq experiments performed in the same cells and the same conditions. Note that the biases are not modelled explicitly from local features of the genome (e.g. G+C content), but implicitly by adjusting the variance of the distribution. Also, the ZINM distribution models the statistical dependence between replicates and thus yields more accurate probabilities than assuming independence.

Given that the genome contains *n* windows, each observation $y_{i}=(y_{i,1},\ldots ,y_{i,r})$ is a vector of *r* read counts (one per replicate), where 1≤i≤n. The associated probability under the ZINM distribution with parameter $\theta =(\pi ,\alpha ,p_{0},\ldots ,p_{r})$ is

$$g(y_{i}|\theta )=\;\{\begin{array}{ll} \pi +(1-\pi )p_{0}^{\alpha } & \text{ if }y_{i}=0\\ (1-\pi )\frac{\Gamma (\alpha +y_{i,1}+\cdots +y_{i,r})}{\Gamma (\alpha )y_{i,1}!\cdots y_{i,r}!}p_{0}^{\alpha }p_{1}^{y_{i,1}}\ldots p_{r}^{y_{i,r}} & \text{ otherwise}.\; \end{array}\;$$

In the definition above, Γ denotes Euler’s gamma function and *y_i_* = 0 stands for the multiple equality $y_{i,1}=\cdots =y_{i,r}=0$. π is the zero-inflation or mixture parameter (the proportion of unmappable windows), α is a shape parameter dictating the distribution of reads and $p_{0},\ldots ,p_{r}$ are probabilities linked by the equality $p_{0}+p_{1}+\cdots +p_{r}=1$ and dictating the average number of reads per window for each replicate.

Discretization is performed by fitting an HMM with ZINM emissions. The HMM has three states corresponding to ‘low’, ‘medium’ and ‘high’ abundance of the given chromatin feature. In many ChIP-seq profiles, the baseline signal shows piece-wise variations of low amplitude but large size (typically 10–100 kb). This will sometimes be the dominant signal and a two-state HMM will identify these blocks instead of the targets. Dedicating two states to fit the baseline is a way to make sure that the ‘high’ state corresponds to the targets of the chromatin feature. In what follows, targets always correspond to the ‘high’ state.

Assuming that *x_i_* takes one of these three values, the log-likelihood of a sequence of states $\left(x_{1},\ldots ,x_{n}\right)$ given the observations $\left(y_{1},\ldots ,y_{n}\right)$ is

$$\log\nu (x_{0})+\log g(y_{0}|x_{0})+\sum_{i=1}^{n}\log Q(x_{i-1},x_{i})+\log g(y_{i}|x_{i}).$$

In the above, *ν* denotes the initial probabilities of the states, *Q* is the 3 × 3 transition matrix of the model, and $g(y_{i}|x_{i})$ is the probability of the emission *y_i_* if the state is *x_i_* (the parameter vector θ depends on the state of the HMM). Discretization amounts to finding the sequence of states and the model parameters that maximize the value above. Zerone achieves this with the Baum-Welch algorithm (Baum and Petrie, 1966), which is a special case of the EM algorithm (Dempster *et al.*, 1977). The emission parameters π and α are state-independent since they represent the baseline distribution of reads in the genomic windows. For this reason they are fitted from the negative control profiles before the Baum-Welch cycles (as a side note, fitting them with the Baum-Welch algorithm slows convergence and sometimes leads to aberrant solutions). The other parameters are state-dependent since they represent the amount of reads per window in each replicate, depending on the abundance of the chromatin feature. Overall, the total number of estimated parameters is 3r+9, where *r* is the number of replicate experiments.

The fitting process resolves conflicts between replicates. Say that a ChIP-seq peak is present in only one of them; the signal will be locally high only for this replicate and low for the others. Because of the conflict, the local log-likelihood will be low for all the possible states but there will still be an optimum that corresponds to the ‘least unlikely’ state. The final call depends on whether the weight of evidence is higher for the presence of the peak or for its absence. If the conflict is strong, the confidence in the final call will be weak, which can lead to a rejection of the profile as a whole if such cases are too frequent (see Section 2.3).

Transition parameters are updated through the forward-backward algorithm (Rabiner, 1989), and emission parameters are updated by solving maximum likelihood equations with the Newton-Raphson method (see Supplementary Material for detail). The state calls are computed by finding the most likely segmentation given the value of the parameters through the Viterbi algorithm (Viterbi, 1967).

### 2.2 ChIP-seq preprocessing

Mapped reads are binned in fixed-step windows (default 300 bp) by their mid-point and PCR duplicates (i.e. reads mapping to the same location in the same orientation) are removed. The window size should not be smaller than the sonication fragment length and it should be set so that there are on average more than 3-4 mapped reads per window. Zerone decompresses on the fly input files compressed by gzip or bgzf (BAM format). There is no upper limit to the number of input files to discretize simultaneously, but there must be at least one negative control and one ChIP-seq experiment (at least two for the quality control to be meaningful, see below).

### 2.3 Quality control

We used a machine learning strategy to identify discretization failures. The true status (success or failure) of experimental ChIP-seq data is not known because success is partly subjective and because there is no gold standard for protein binding in live cells. We prepared an experimental dataset where we labelled the output of Zerone as positive (success) or negative (failure) based on empirical criteria (see associated Docker image for detail). The definition of success is thus subjective, but the training is performed on representative data.

We discretized 96 replicated ChIP-seq experiments together with their respective input control (see associated Docker image). Based on visual inspection and on the available literature, we determined that discretization was successful in 91 cases that consituted the positive examples of our training set. The most common cases of poor data quality in ChIP-seq correspond to low signal-to-noise ratio (e.g. when the antibody is not specific), and lack of reproducibility between replicates (e.g. when samples are swapped). We created 91 negative cases obtained by discretizing controls without immunoprecipitation, or nonreplicate profiles (e.g. by treating CTCF and Pol2 profiles as replicates). Thus, a total of 182 cases (91 positive and 91 negative) were used to build a balanced dataset. We extracted 5 features from the output of Zerone to train a classifier: the transition matrix entry $Q_{2,0}$ (indicating the size of the targets), the minimum value of the ratios $p_{2}(2)/p_{2}(1),\ldots ,p_{r}(2)/p_{r}(1)$ (indicating the signal to noise ratio), the amount of targets, the fraction of variance explained by the discretization and the correlation between replicates.

**Fig. 2.**
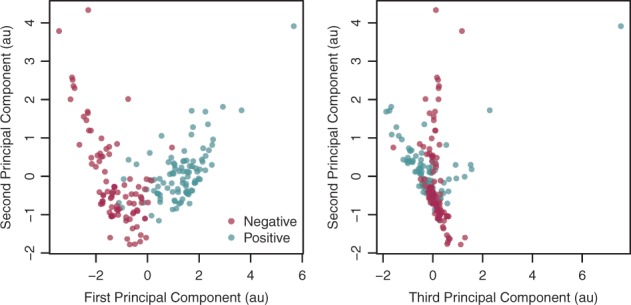
Scaled Principal Component Analysis of the training dataset (color version of the figure available online). Each symbol represents a discretization performed by Zerone. The five features extracted from each discretization are projected onto the first three principal components. The two groups overlap, which creates an ambiguous zone where failures and successes are hard to distinguish (Color version of this figure is available at *Bioinformatics* online.)

To separate the points in the feature space (Fig. 2), we used a Support Vector Machine (SVM, Chang and Lin, 2011; Meyer *et al.*, 2014) with a radial basis function kernel, as they allow nonlinear classification, are fast to train and require only two hyperparameters to be fitted. We trained the SVM and selected the hyperparameters that maximized the prediction performance on test sets using a 10-fold cross-validation scheme. The average prediction accuracy on these sets was 95%. Unlike quality control methods based on individual peaks (such as the IDR for instance) the quality control implemented in Zerone is ‘all-or-none’, i.e. the profile is rejected or accepted as a whole.

### 2.4 Benchmark datasets and conditions

To train the SVM, we used all the ChIP-seq fastq files produced by the ENCODE Consortium on the human leukemia cell line K562. Reference assembly UCSC hg19 was used throughout. The data was downloaded from the repository http://hgdownload.cse.ucsc.edu/goldenPath/hg19/encodeDCC in November 2013. We mapped all the raw reads on the human genome with GEM (gem-mapper version 1.376 beta, gem-indexer version 1.423 beta, Marco-Sola *et al.*, 2012), using the options—unique-mapping and -q ignore. We converted the mapped files to SAM format with gem-2-sam version 1.423 beta.

To compare Zerone to other discretizers, we analyzed two different ChIP-seq datasets: CCCTC-binding factor (CTCF) and tri-methylated histone H3 at lysine 36 (H3K36me3). Each dataset consists of a mock or input profile without immunoprecipitation and two replicate ChIP-seq profiles. The ENCODE accession numbers for these four datasets are ENCSR000DWE and ENCSR000DWD respectively. Table 1 gives a global overview of the datasets used for benchmarking.

**Table 1.** Summary statistics of the datasets used for benchmarking

Dataset	Read size	Sequencing depth	Mapped reads
Input	36	18 123 856	18 064 246
CTCF^(1)^	36	32 740 518	15 698 068
CTCF^(2)^	36	27 953 212	12 971 023
H3K36me3^(1)^	36	18 174 968	13 847 015
H3K36me3^(2)^	36	18 495 290	14 419 200

Numbers in parentheses are used to distinguish replicates.

We tested MACS callpeak version 2.1.0.20140616, BayesPeak version 1.20.0 and JAMM version 1.0.7rev1. All tests were performed on an 8-core Intel Xeon E5606 machine with 48 GB of DDR3-RAM at 1333 MHz. All programs were run on a single core with the recommended options. Specifically, on the H3K36me3 dataset, JAMM was run with the option -r region and MACS with the -broad option. For the rest of the datasets and programs, the default options were used. When using IDR, we ran MACS with a relaxed *q*-value cutoff set to 0.05 to obtain a higher number of peaks. For JAMM, we took the top ranking 300 000 peaks as input for IDR. The IDR analysis proper was conducted using the R script batch-consistency-analysis.r, available at https://sites.google.com/site/anshulkundaje/projects/idr. For MACS, peaks scoring lower than 0.05 were kept. For JAMM, the top *n* peaks in the joint discretization were kept, where *n* is the number of peaks scoring lower than 0.05 in separate discretizations. For the CTCF benchmark, all peaks were resized to 500 bp from the center of the window.

The CTCF motif was obtained from the JASPAR database version 5.0_ALPHA (motif ID MA0139.1, Mathelier *et al.*, 2014). Subsequently we used FIMO (Grant *et al.*, 2011) from the MEME suite version 4.10.1 (Bailey *et al.*, 2009) to identify and map CTCF motif occurences in the human genome. The positions of Transcription Start Sites (TSS) were extracted from the knownGene table of the UCSC Genes annotation (Karolchik *et al.*, 2004). RNA counts in section 3.2.2 were obtained from the ENCODE dataset EH000163 (already mapped bigWig files).

The quality control of Zerone was compared to a method based on IDR to flag replicates with low consistency or low quality (described in https://sites.google.com/site/anshulkundaje/projects/idr). Briefly, this method uses the script batch-consistency-analysis.r to perform pairwise comparisons between all the replicates to check that the number of reproducible peaks is similar between them. The same comparisons are also performed between random splits of each replicate, and between random splits of a pooled profile. Experiments that do not satisfy minimal criteria are flagged as faulty.

A full replay of the benchmark including all the necessary datasets and scripts is available from the associated Docker image.

## 3 Results

### 3.1 Automatic quality control

The most novel feature of Zerone is an embedded automatic quality control step taking place after the discretization. It not only ensures that the discretization is sensible, but also that the replicates are similar to each other and that the ChIP-seq profiles are not too similar to the mock controls. Our approach is based on the idea that discretizations from overly noisy or divergent profiles should have a signature that can be picked up by a specially trained classifier.

We identified five summary statistics that characterize the quality of the discretization and trained an SVM to recognize failures. We thus obtained a classifier able to identify a failed discretization with 95% accuracy (see Section 2.3).

**Fig. 3.**
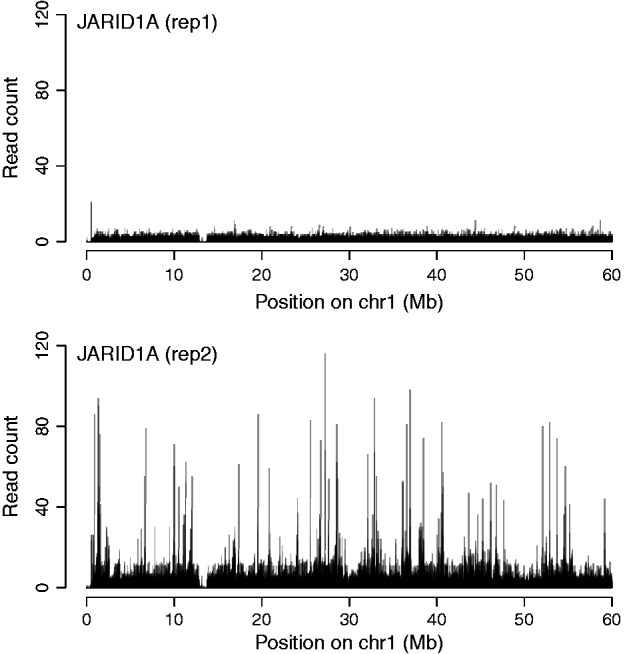
ChIP-seq profiles of JARID1A in H1 ES cells (300 bp windows). The first replicate is not similar to the second, and it does not contain any target

This feature is essential for high throughput automatic pipelines. For instance, when discretizing ENCODE ChIP-seq profiles obtained in human H1 ES cells, we noticed that the lysine-demethylase JARID1A did not pass the quality control. Further investigation immediately revealed the nature of the issue. In one of the replicates, the signal is lacking entirely, as if the immunoprecipitation failed (Fig. 3). Once aware of the issue, users can handle it properly (for instance, by discarding the protein or by working with a single replicate). Without automatic quality control, the low quality of the first replicate would have been missed.

Low quality profiles can also be detected using IDR. We ran either Zerone or MACS followed by IDR-based flagging on a test set of 30 chromatin features from H1 ES cells. The agreement between the two methods was 85% (see Section 2.4 and associated Docker image). We excluded five cases where either MACS or IDR failed to run. Among the 8 cases of disagreement, Zerone accepted three and rejected five, suggesting that it is slightly more stringent than MACS + IDR. It is important to note that the quality control of Zerone is more general than IDR-based flagging since it applies to all types of signal without upper limit on the number of replicates. In contrast, IDR is a tool for pairwise comparisons and it does not apply to profiles with broad domains. Also note that IDR-based flagging in itself can represent a significant computational burden when there are many replicates or many targets sites (Section 3.3 shows some examples of the computational cost of IDR).

### 3.2 Accuracy

**Fig. 4.**
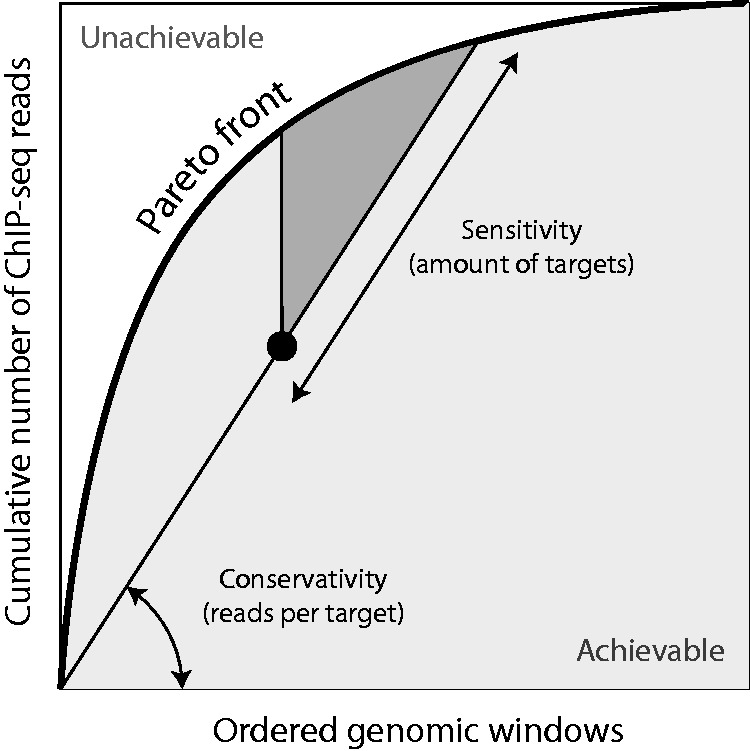
Graphical representation of discretizations. Genomic windows of the ChIP-seq profile are ordered by decreasing amount of reads on the *x* axis, and the cumulative amount of reads is plotted on the *y* axis. This line forms a Pareto front representing the maximum number of reads for a given number of windows. Discretizations are represented as a single point on this plane (black disc) whose coordinates are the number of targets and the total number of reads covered by the targets. The dark triangle represents discretizations that are more conservative and discover more targets

The purpose of discretizers is to identify the targets of a transcription factor or a histone mark, i.e. the sites of the genome where it is present. Intuitively, good discretizers capture a large fraction of the ChIP-seq signal within few targets. The number of targets and the amount of reads they represent are therefore critical characteristics of a discretization. Unfortunately, there is no gold standard to estimate the trade-off between false positives and false negatives in ChIP-seq experiments, and thus there is no objective way to rank discretizers (Szalkowski and Schmid, 2011). However, we can compare them with a partial order as follows: when arranging genomic windows from high to low amount of ChIP-seq reads, the cumulative number of reads forms a Pareto front. It represents the largest amount of reads that can be captured by the given amount of targets (Fig. 4). A discretization can be represented as a point of this plane. By construction, no discretization can lie on the left of the Pareto front, and those on the front represent an optimum. Others are suboptimal, since it is possible to capture more reads with the same amount of targets (or the same amount of reads with less targets).

We benchmarked Zerone against three ChIP-seq discretizers. We included MACS as the standard method for ChIP-seq peak calling, BayesPeak because it is powered by an HMM with ZINB emissions similar to the model implemented in Zerone, and JAMM because it can perform joint discretization of experimental replicates. We used datasets of similar size that represent two major types of ChIP-seq signal (Table 1). The CTCF signal consists of sharp peaks at the transcription factor binding site and the H3K36me3 signal consists of broad domains.

**Fig. 5.**
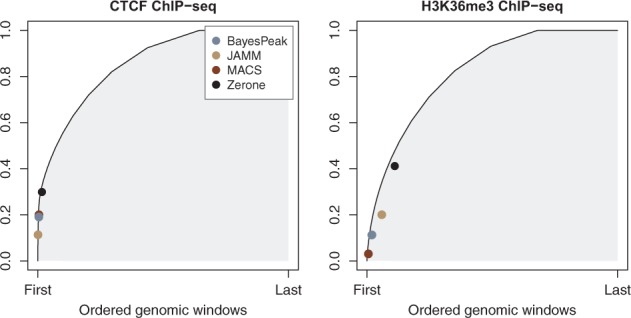
Characteristics of the discretizations for different programs (color version of the figure available online). The representation is obtained as shown on Figure 4. The CTCF discretization produced by Zerone is close to the Pareto front. For H3K36me3, the discretization is off the Pareto front, but it is more sensible than the others

This representation reveals that discretizing the CTCF profile yields similar outputs regardless of the software (Fig. 5, left panel). On the other hand, discretizing the H3K36me3 profile yields very distinct outputs. In all the cases, Zerone produces the discretization capturing the most reads. For CTCF it lies on the Pareto front. For H3K36me3, it lies somewhat off the Pareto front, but at a sensible location. As detailed below, H3K36me3 is deposited on transcribed genes (Kimura, 2013; Pokholok *et al.*, 2005) so the coverage of targets should be higher than for transcription factors and for other profiles with sharp peaks. Taken together, these results show that Zerone produces discretizations that are sensitive and adapted to the profile being discretized.

#### 3.2.1 Identification of CTCF binding sites

CTCF binds a 20 bp consensus sequence that is highly conserved in vertebrates. In humans, nearly 80% of the CTCF binding sites contain the consensus motif (Kim *et al.*, 2007). In order to determine the capacity of the different tools to call peaks of CTCF binding, we compared the discretized profiles against a reference dataset containing the positions of 85 690 occurences of the CTCF motif (see Section 2.4).

Table 2 shows that for most tools, the *F*_1_ score (the harmonic mean of precision and recall) is between 0.34 and 0.41. The exception is JAMM, achieving significantly higher precision than the other tools at the cost of recall. On this dataset, the performance of Zerone is fair, with a good balance between precision and recall.

**Table 2.** Performance on the CTCF motifs dataset

Software	Total	Motif	Precision	Recall	*F*_1_ score
BayesPeak^(1)^	45 229	25 223	0.56	0.32	0.40
BayesPeak^(2)^	45 192	23 637	0.52	0.30	0.38
JAMM	11 046	8777	0.79	0.11	0.20
MACS^(1)^	48 341	26 475	0.55	0.33	0.41
MACS^(2)^	41 048	23 498	0.57	0.30	0.39
MACS^(1)^ + IDR	25 080	17 530	0.70	0.22	0.34
MACS^(2)^ + IDR	25 080	17 527	0.70	0.22	0.34
Zerone	54 315	25 324	0.47	0.32	0.38

The numbers in parentheses indicate the results on the two replicates separately. True and false positives are defined as peaks with and without a CTCF motif, respectively. Total: number of peaks found by the program. Motif: subset of those containing at least one CTCF motif. Precision: Motif divided by Total. Recall: number of motifs covered by peaks divided by the number of motifs in the genome. *F*_1_ score: harmonic mean between Precision and Recall.

The numbers in parentheses indicate the results on the two replicates separately.

#### 3.2.2 H3K36me3-enriched domains on active genes

There is no consensus sequence to determine the location of histone modifications. However, it is known that the bodies of active genes are enriched in H3K36me3 (Kimura, 2013; Pokholok *et al.*, 2005). Therefore, the genes that contain peaks or windows determined as enriched in H3K36me3 by the different discretizers should be more expressed than the background.

**Fig. 6.**
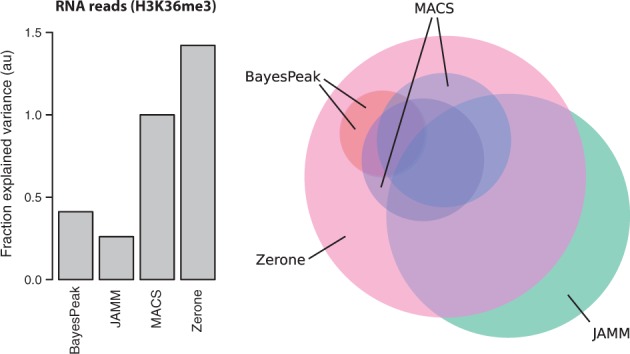
Left panel: quality of the H3K36me3 discretization. Each bar represents the relative fraction of variance in RNA reads explained by the discretization of H3K36me3. The value for MACS was set to 1.0 and all other values were scaled accordingly. Right panel: Overlap between H3K36me3 targets (color version of the figure available online). Each circle represents the H3K36me3 targets identified by a program. The size of the circles is proportional to the coverage of the targets, and their overlap approximates the amount of targets shared by the programs. Note that the discretizations made by BayesPeak overlap almost completely and are indistinguishable in this representation. Also note that JAMM is used ‘unthresholded’, as IDR is not recommended for broad signal (Color version of this figure is available at *Bioinformatics* online.)

We benchmarked the quality of the discretization with expression data obtained in the same cell. We used the number of RNA reads as a response variable and computed the amount of variance explained by the discretized profile of H3K36me3. The discretization produced by Zerone is the best predictor of expression (Fig. 6, left panel). It is also the one with highest coverage (Fig. 5, right panel), which shows that the increased number of targets does not come at the cost of accuracy. On the contrary, the high coverage of H3K36me3 is confirmed by expression data.

A Venn diagram gives a graphical overview of the relationships between the discretizations (Fig. 6, right panel). Zerone finds most of the targets detected by the other discretizers, while discovering enriched windows not found by the others.

### 3.3 Speed and memory footprint

**Fig. 7.**
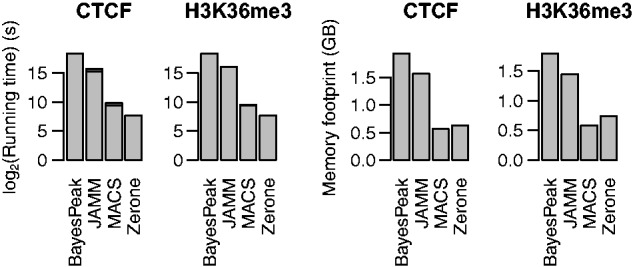
Running times and peak memory footprint of the discretizers on the four ChIP-seq datasets. For programs that only allow single-profile discretization (i.e. BayesPeak and MACS), mean values (not the sum) are shown. The bars in dark grey represent the total running time when IDR is also computed (for JAMM and MACS). Note that the logarithmic scale misrepresents the relative fraction of time spent on each task

We compared the running times of the different programs on discretizing the four datasets used above, containing 18–32 million reads (Table 1). The results were similar between experiments. Zerone was consistently the fastest tool, with a running time of around 5 min (Fig. 7, top row). The advantage is marginal over MACS, which ran for around 10 min, but it is substantial over JAMM and BayesPeak which ran in hours or in days, respectively. Post-processing the output with IDR did not significantly increase the running time of MACS, but it did so for JAMM, increasing the total running time by a few hours (Fig. 7, dark grey bars). The results for peak memory usage were variable between experiments (Fig. 7, bottom row). MACS achieved the best performance with a memory footprint around 0.5 GB, followed by Zerone around 0.7 GB. BayesPeak and JAMM each used more than 1.5 GB. The memory usage of Zerone does not depend on the sequence depth of the experiment, but solely on the number of replicates and the total number of windows in the genome. As an example, for the human genome at 300 bp resolution, the memory footprint of Zerone is expected to be around 500 MB + 40 MB per replicate.

The benchmark is partly confounded by the fact that Zerone discretizes multiple profiles simultaneously. This makes a difference for pipelines where all files have to be processed in parallel with the minimum amount of resources. In this benchmark, Zerone processed twice as many files per run as MACS. Per processed file, Zerone is thus four times faster than MACS while using 30% less memory.

## 4 Discussion and conclusions

Zerone was developed ground up for scalibility and throughput. The result is a tool with competitive performance (Fig. 7). Part of the speed is due to hashing methods that dramatically cut down the computation time during the Baum-Welch cycles. Zerone also rests on sound statistical bases. Theoretical arguments and experimental observations suggest that the Zero-Inflated Negative Multinomial distribution is appropriate to model ChIP-seq data (Fig. 1). This gives Zerone good specificity and sensitivity for very different profiles (Fig. 5).

Zerone also proposes an original solution to the problem of data heterogeneity. Firstly, the statistical model is fitted in order to harmonize the replicates and solve conflicts by maximum likelihood. Secondly, automatic quality control is performed after the discretization. The principle of this step is somewhat similar to anomaly detection. Note that control profiles play a key role in the process. In order to evaluate the quality of the discretization, Zerone implicitly assumes that the user has provided controls that properly capture systematic biases such as batch effects, mappability and copy number variations.

The quality control implemented in Zerone goes beyond the IDR (Li *et al.*, 2011) in several ways. Zerone measures the quality of the discretization and not only the consistency between replicates. Also, the quality control of Zerone is neither limited to a specific type of profile (e.g. sharp peaks), nor to a preset number of replicates. Finally, issuing an ‘all-or-none’ call about the discretization is better practice than silently ignoring the regions that differ between experiments (see e.g. Fig. 3).

Here, we also introduced a way to compare discretizers with an intuitive graphical representation (Fig. 4). On this plane, the coordinates of a discretization indicate the number of targets (or occupancy) and the amount of reads captured by these targets. The Pareto front captures the inherent trade-off between sensitivity and specificity in the problem of discretizing ChIP-seq profiles. Points on this line that are close to the bottom-left corner represent discretizations with high amount of reads per target (most specific) and points that are close to the top-right corner represent discretizations with many targets (most sensitive). The Pareto front also highlights an unachievable region whose shape depends on the structure of the signal, i.e. on the feature being discretized (Fig. 4). One of the challenges of discretizing ChIP-seq profiles is to find algorithms that perform well in all the cases.

In practice, the specificity of a discretizer is unknown because the biological truth remains hidden. However, we can decide whether a discretizer is more or less conservative than another by measuring the amount of reads per target. This characteristic may be a matter of choice, and is usually tacit in the case of ChIP-seq discretizers. Out of two equally conservative discretizers, one may be more sensitive, i.e. discover more targets. The merit of the representation introduced here is to highlight these characteristics and to guide users when choosing the most appropriate tool for their need. Note, however, that the number of reads per window is not the only criterion for calling targets, so there are cases where a discretization lying far from the front may be preferable to one lying closer to it.

This representation naturally suggests a naive approach to discretize ChIP-seq profiles. Indeed, one could sort the genomic windows by decreasing amount of ChIP-seq reads and call ‘target’ any window above a chosen threshold. While this method would only produce discretizations on the Pareto front, adjusting the threshold to the conditions would be challenging for lack of an underlying model. This is one of the major strengths of Zerone: the statistical model automatically adjusts conservativity and sensitivity in a sensible way.

In summary, the good performance of Zerone on different classes of profiles, combined with the automatic quality control meet the needs for general and robust ChIP-seq tools.

## Supplementary Material

Supplementary Data
