# Supplementary material for: Zerone: a ChIP-seq discretizer for multiple replicates with built-in quality control
Source: Bioinformatics. 2016 Jun 10;32(19):2896–902. doi: 10.1093/bioinformatics/btw336 (PMC5039920; doi:10.1093/bioinformatics/btw336)
Supplement: Supplementary Data [file supp_32_19_2896__index.html]

Zerone: a ChIP-seq discretizer for multiple replicates with built-in quality control — Zerone: a ChIP-seq discretizer for multiple replicates with built-in quality control — Supplementary Data 

# Zerone: a ChIP-seq discretizer for multiple replicates with built-in quality control

## Supplementary Data

files

- Supplementary Data - pdf file
