# Supplementary material for: Zerone: a ChIP-seq discretizer for multiple replicates with built-in quality control
Source: Bioinformatics. 2016 Jun 10;32(19):2896–902. doi: 10.1093/bioinformatics/btw336 (PMC5039920; doi:10.1093/bioinformatics/btw336)
Supplement: Supplementary Data [file supp_btw336_Supplementary_information.pdf]

Supplementary information

Zerone: a ChIP-seq discretizer for multiple replicates with built-in quality control

Guillaume Filion and Pol Cuscó

October 22, 2015

We start with some generalities about Hidden Markov Models and then derive a model targeted to ChIP experiments with replicates.

1 Hidden Markov Models

We will consider only discrete Hidden Markov models (HMMs) and will simply refer to them as Hidden Markov model, without mention of the term 'discrete' for simplicity. HMMs are defined by

1. a set S of m states numbered from 1 to m,
2. an initial state probability distribution ν, which gives the probabilities that the system is initially in state i,
3. an $m \times m$ transition matrix Q which contains the probabilities $Q(i, j)$ that the system goes from state i to state j,
4. m distributions denoted g_i ($i = 1, \dots, m$), which give the emission probabilities in the different states.

1.1 The HMM formalism applied to ChIP-seq

The output of ChIP-seq experiments consists of genomic profiles that can be thought of as ordered windows of equal size. Each window is associated to

a certain number of read counts coming from different replicate experiments or negative controls.

HMMs are particularly adapted to this framework. The read counts associated to each window can be thought of as the observable emissions, whereas the unobservable states can be thought of the possible processes ongoing on those windows. Typically, the states may correspond to the events "the protein of interest is bound on the window" and "the protein of interest is not bound on the windown". There may be more states, and they do not have to correspond to a protein binding event (most notably, they can correspond to the present of some histone modifications).

The rest of this section pertains to general HMMs and will not make any hypothesis regarding the nature of the states and the emissions, however it can be useful for the intuition to think about states are chromatin states, and emissions as read counts.

1.2 The Forward-Backward algorithm

For a sequence of emissions $y_0, \ldots, y_n$, the likelihood of the state sequence $i_0, \ldots, i_n$ is proportional to

$$\nu(i_0) g_{i_0}(y_0) \prod_{k=1}^{n} Q(i_{k-1}, i_k) g_{i_k}(y_k).$$

By summing over all possible combinations of states, we obtain the normalizing constant L_n such that

$$L_n = \sum_{i_0 \in S, \ldots, i_n \in S} \nu(i_0) g_{i_0}(y_0) \prod_{k=1}^{n} Q(i_{k-1}, i_k) g_{i_k}(y_k). \tag{1}$$

We denote $\phi_{k|n}(i)$ the probability that the system is in state i at time k given the emissions $y_0, \ldots, y_n$. If we call $S_n(k, i)$ the set of n-tuples $(i_0, \ldots, i_n)$ such that $i_k = i$, the value of $\phi_{k|n}(i)$ comes as

$$\phi_{k|n}(i) = \frac{1}{L_n} \sum_{(i_0, \ldots, i_n) \in S_n(k,i)} \nu(i_0) g_{i_0}(y_0) \prod_{l=1}^{n} Q(i_{l-1}, i_l) g_{i_l}(y_l).$$

We now introduce $\alpha_k(i)$ the probability that the system is in state i at time k given the emissions $y_0, \ldots, y_k$, and the $\beta_{k|n}(\cdot)$ the numerical function such that $\phi_{k|n}(i) = \alpha_k(i) \beta_{k|n}(i)$.

$$\alpha_k(i) = \frac{1}{L_k} \sum_{i_0=1}^{m} \cdots \sum_{i_{k-1}=1}^{m} \nu(i_0) g_{i_0}(y_0) \prod_{l=1}^{k-1} Q(i_{l-1}, i_l) g_{i_l}(y_l) Q(i_{k-1}, i) g_i(y_k)$$

$$\beta_{k|n}(i) = \frac{L_k}{L_n} \sum_{i_{k+1}=1}^{m} \cdots \sum_{i_n=1}^{m} Q(i, i_{k+1}) g_{i_{k+1}}(y_{k+1}) \prod_{l=k+2}^{n} Q(i_{l-1}, i_l) g_{i_l}(y_l)$$

To preserve the equality $\phi_{k|n}(i) = \alpha_k(i)\beta_{k|n}(i)$ for every k, we set by definition $\beta_{n|n}(i) = 1$. From the equations above, we draw the following recursive equations:

$$\alpha_k(i) = \frac{L_{k-1}}{L_k} \sum_{j=1}^{m} \alpha_{k-1}(j) Q(j, i) g_i(y_k) \tag{2}$$

$$\beta_{k|n}(i) = \frac{L_k}{L_{k+1}} \sum_{j=1}^{m} Q(i, j) g_j(y_{k+1}) \beta_{k+1|n}(j). \tag{3}$$

Equations (2) and (3) are the basis of the Forward-Backward algorithm to compute $\phi_{k|n}(i)$. The terms $\alpha_k(i)$ can be recursively computed from $k = 0$ to $k = n$ with equation (2), and the terms $\beta_{k|n}(i)$ can be computed from $k = n - 1$ to $k = 0$ with equation (3). The terms $\phi_{k|n}(i)$ are then found as the product $\alpha_k(i)\beta_{k|n}(i)$.

We now turn to the term $\phi_{k-1,k|n}(i, j)$, which is by definition the probability that the system is in state i at time $k - 1$ and in state j at time k given $y_0, \ldots, y_n$. If we call $S_n(k, i, j)$ the set of n-tuples $(i_0, \ldots, i_n)$ such that $i_{k-1} = i$ and $i_k = j$, we get

$$\begin{aligned} \phi_{k-1,k|n}(i, j) &= \frac{1}{L_n} \sum_{(i_0,\ldots,i_n) \in S_n(k,i,j)} \nu(i_0) g_{i_0}(y_0) \prod_{l=1}^{n} Q(i_{l-1}, i_l) g_{i_l}(y_l) \\ &= \frac{L_{k-1}}{L_k} \alpha_{k-1}(i) Q(i, j) g_j(y_k) \beta_k(j). \end{aligned} \tag{4}$$

When the $\alpha_k(i)$ and the $\beta_{k|n}(i)$ have been computed by the Forward-Backward algorithm, we also have access to the $\phi_{k-1,k|n}(i, j)$ by using formula (4).

1.3 The Baum-Welch algorithm

The Baum-Welch algorithm is the special case of the EM algorithm applied to HMMs. Let us consider the general case of the triplet (X, Z, θ) where the variable X is observed, Z is not observed, and θ is the set of parameters of the distribution of (X, Z). The full likelihood $\mathcal{L}_0(X, Z, \theta)$ cannot be computed because the value of Z is unknown.

To find the value of θ that maximizes the full likelihood, we introduce an iterative procedure where the values of the parameter are updated upon each iteration. The current value of θ is noted $\theta^{(t)}$, and we compute the expected complete log-likelihood $\mathcal{Q}(\theta|\theta^{(t)})$ assuming the current value of θ (note the difference between the intermediate quantity of the EM $\mathcal{Q}$ and the transition matrix Q).

$$\mathcal{Q}(\theta|\theta^{(t)}) = E_{Z|X,\theta^{(t)}} \left\{ \log \mathcal{L}_0(X, Z, \theta^{(t)}) \right\}$$

This computation is called the E-step. The notations mean that the expectation is taken over the variable Z, assuming that it is conditional on the observed values of X and that the parameters of the distribution are given by $\theta^{(t)}$. The E-step is followed by the M-step, in which $\theta^{(t+1)}$ is set to the value of θ that maximizes $\mathcal{Q}(\theta|\theta^{(t)})$.

In the case of HMMs, the variable that is not observed is the sequence of states. The set of parameters $\theta^{(t)}$ represents the transition probabilities (the matrix Q) and the parameters of the m distributions of the emissions.

The log-likelihood of the state sequence $(i_0, \ldots, i_n)$ is

$$\log \nu(i_0) + \sum_{k=1}^{n} \log Q(i_{k-1}, i_k) + \sum_{k=0}^{n} \log g_{i_k}(i_k, \theta).$$

The addition of θ to the terms above emphasizes that they depend on the value of the parameters. To compute $\mathcal{Q}(\theta|\theta^{(t)})$, we need to take the expectation of the above over the state sequence conditionally on $y_0, \ldots, y_n$ and assuming that the parameters are given by $\theta^{(t)}$.

$$
\begin{aligned}
\mathcal{Q}(\theta|\theta^{(t)}) = E_{\theta^{(t)}} \left\{ \log \nu(i_0) \middle| y_0, \ldots, y_n \right\} + \\
\sum_{k=1}^{n} E_{\theta^{(t)}} \left\{ \log Q(i_{k-1}, i_k) \middle| y_0, \ldots, y_n \right\} + \\
\sum_{k=0}^{n} E_{\theta^{(t)}} \left\{ \log g_{i_k}(y_k, \theta) \middle| y_0, \ldots, y_n \right\}
\end{aligned}
\tag{5}
$$

In practice, the first term of (5) will often not depend on θ so it will not contribute to the evaluation. The third term can be rewritten as

$$
\sum_{k=0}^{n} \sum_{i=1}^{m} \phi_k(i) \log g_{i_k}(y_k, \theta).
$$

This term depends on the emission probabilities, and nothing can be said about it in general terms because they differ between different models. But the second term depends only on the transition probabilities, which are present in every HMM, and it can be solved in general. First we notice that

$$
\begin{aligned}
& E_{\theta^{(t)}} \left\{ \log Q(i_{k-1}, i_k) \middle| y_0, \ldots, y_n \right\} = \\
& E_{\theta^{(t)}} \left\{ \sum_{i=1}^{m} \sum_{j=1}^{m} 1_{\{(i_{k-1}, i_k) = (i,j)\}} \log Q(i,j) \middle| y_0, \ldots, y_n \right\} = \\
& \sum_{i=1}^{m} \sum_{j=1}^{m} E_{\theta^{(t)}} \left\{ 1_{\{(i_{k-1}, i_k) = (i,j)\}} \middle| y_0, \ldots, y_n \right\} \log Q(i,j)
\end{aligned}
$$

Remember that by definition $E_{\theta^{(t)}} \left\{ 1_{\{(i_{k-1}, i_k) = (i,j)\}} \middle| y_0, \ldots, y_n \right\}$ is $\phi_{k-1,k}(i,j)$, so that we can rewrite the second term of (5) as

$$
\sum_{k=1}^{n} \sum_{i=1}^{m} \sum_{j=1}^{m} \phi_{k-1,k}(i,j) \log Q(i,j).
$$

The values of $\phi_{k-1,k}(i,j)$ are computed during the E-step by the Forward-Backward algorithm. The terms $Q(i,j)$ are part of θ and are thus updated during the M-step. By using Lagrange multipliers, we can show that the update values are

$$Q(i,j)^{(t+1)} = \frac{\sum_{k=1}^{n} \phi_{k-1,k}(i,j)}{\sum_{k=1}^{n} \sum_{l=1}^{m} \phi_{k-1,k}(i,l)}.$$

To complete the Baum-Welch algorithm, we need to compute the last term of (5), which requires making a model for the emissions.

2 Zero-Inflated Negative Multinomial

The Baum-Welch algorithm provides a general framework to estimate the transition probabilities and the emission parameters. However, the detail of the estimation depends on the emission model. Here we give some general results about the negative multinomial and the zero-inflated negative multinomial distributions that will be useful to setup a model for emissions in ChIP-seq experiments.

2.1 The Gamma-Poisson process

In what follows, y is a non negative integer (an element of $\mathbb{N}$). Let Y be a discrete random variable distributed as a Poisson distribution with parameter λ. The probability that Y is equal to y is by definition

$$P(Y = y) = e^{-\lambda} \frac{\lambda^y}{y!}. \tag{6}$$

Let us now assume that λ is itself a random variable, such that the above equality is actually $P(Y = y|\lambda)$. If λ is independent of Y and has a Gamma distribution with parameters α and β, the joint distribution of Y and λ is the product of the two distributions, that is

$$e^{-\lambda} \frac{\lambda^y}{y!} \frac{1}{\Gamma(\alpha)\beta^\alpha} e^{-\lambda/\beta} \lambda^{\alpha-1}.$$

The marginal distribution of Y, *i.e.* $P(Y = y)$, is found by integrating the equality above over λ.

$$
\begin{aligned}
P(Y=y) &= \frac{1}{\Gamma(\alpha)\beta^{\alpha}y!}\int_0^{+\infty} e^{-\lambda(1+1/\beta)}\lambda^{\alpha+y-1}d\lambda \\
&= \frac{\Gamma(\alpha+y)}{\Gamma(\alpha)\beta^{\alpha}(1+1/\beta)^{\alpha+y}y!} \\
&= \frac{\Gamma(\alpha+y)}{\Gamma(\alpha)y!}\left(\frac{1}{1+\beta}\right)^{\alpha}\left(\frac{\beta}{1+\beta}\right)^{y}. \qquad (7)
\end{aligned}
$$

Equation (7) is the expression of the negative binomial distribution, with one of the many possible parametrizations. We will refer to this distribution as a negative binomial with parameters $(\alpha, 1/(1+\beta))$.

The Gamma-Poisson process can describe many phenomena because of the flexibility of the Gamma distribution. The α parameter of the Gamma distribution is often referred to as the "shape" parameter. For negative values of *alpha*, the distribution has a singularity at 0, whereas for positive values, the distribution has a single "bump". The β parameter is often referred to as the "scale" parameter because it represents a stretching of the curve along the x-axis that can fit different variances. As a result, the Gamma distribution is a good choice for almost every continuous distribution with positive values and a single mode. Combined to the Poisson distribution, it allows to fit almost every discrete distribution with positive values.

2.2 The negative multinomial distribution

We now introduce the case of r Poisson variables that are conditionally independent given λ. Intuitively, this means that λ is drawn at random first, which sets the parameter of the r Poisson distributions; the Poisson variables are then drawn from their respective distribution independently of each other. In other words, the conditional distribution can be written as follows

$$
P(Y_1 = y_1, \dots, Y_r = y_r|\lambda) = e^{-\gamma_1\lambda}\frac{(\gamma_1\lambda)^{y_1}}{y_1!}\dots e^{-\gamma_r\lambda}\frac{(\gamma_r\lambda)^{y_r}}{y_r!}. \qquad (8)
$$

Multiplying by the density of λ and integrating as above, the marginal distribution of the vector $(Y_1, \dots, Y_r)$ comes as

$$P(Y_1 = y_1, \ldots, Y_r = y_r) = \frac{\Gamma(\alpha + y_1 + \ldots + y_r)}{\Gamma(\alpha) y_1! \ldots y_r!} p_0^{\alpha} p_1^{y_1} \ldots p_r^{y_r}, \text{ where} \tag{9}$$
$$p_0 = \frac{1/\beta}{1/\beta + \gamma_1 + \ldots + \gamma_r}, \text{ and}$$
$$p_i = \frac{\gamma_i}{1/\beta + \gamma_1 + \ldots + \gamma_r}, \text{ for } i = 1, \ldots, r.$$

This distribution is called the negative multinomial, which the negative binomial is a special case of (for $r = 1$). We will refer to it is as a negative multinomial with parameters $(\alpha, p_1, \ldots, p_r)$. It can be interpreted as the observations of a Gamma-Poisson process, where a common λ is drawn from a Gamma distribution, and r variables are drawn from independent Poisson distributions with parameters $\gamma_i \lambda$ ($1 \leq i \leq r$). The variables $Y_1, \ldots, Y_r$ are independent contionally on λ, but in section 2.3 we prove that they are never unconditionally independent.

The negative multinomial distribution has an alternative interpretation that sometimes proves useful. Suppose an urn contains black balls and balls of r different colors in respective proportions $p_0, p_1, \ldots, p_r$, such that $p_0 + p_1 + \ldots + p_r = 1$. If we draw balls with replacement from the urn until a black ball is drawn for the k-th time, the probability that the counts for the balls of each color are $(y_1, \ldots, y_r)$ is

$$\binom{k - 1 + y_1 + \ldots + y_r}{(k-1), y_1, \ldots, y_r} p_0^k p_1^{y_1} \ldots p_r^{y_r} = \frac{\Gamma(k + y_1 + \ldots + y_r)}{\Gamma(k) y_1! \ldots y_r!} p_0^k p_1^{y_1} \ldots p_r^{y_r}.$$

This is formula (9), where α has been replaced by k. The negative multinomial distribution is thus a generalization of the drawing process described above. From the ball and urn interpretation, we get that the observed counts $(y_1, \ldots, y_r)$ are twice smaller for a twice larger value of p_0 or for a twice smaller value of α.

2.3 Marginal distributions

To compute the marginal distributions of $(Y_1, \ldots, Y_r)$, we could sum over (9), but it is simpler to sum over (8) and then multiply by the density of λ and integrate. The sum of (8) over all indices distinct from $s \leq r$ is a Poisson

term of the form of (6) therefore, integrating over λ yields an expression similar to (7), namely

$$
\begin{aligned}
P(Y_s = y) &= \frac{\Gamma(\alpha + y)}{\Gamma(\alpha) y!} p_0^{*\alpha} p_s^{*y}, \text{ where} \\
p_0^* &= \frac{1/\beta}{1/\beta + \gamma_s}, \text{ and} \\
p_s^* &= \frac{\gamma_i}{1/\beta + \gamma_s}.
\end{aligned}
$$

Not surprisingly, we obtain a negative binomial distribution. More interestingly though, the parameters of this distribution are linked to the previous parameters by the equality $p_s^*/p_0^* = p_s/p_0$. This equality comes in handy to recompute the parameters of the negative multinomial distribution when variables are added or removed.

In the light of the analogy with balls in an urn, this result is not surprising. The marginal distribution corresponds to the same process where some colors are removed. The proportion of the remaining colors change, but their relative ratios do not.

To illustrate the use of this equality, we show with $r = 2$ that the marginal variables of a negative multinomial distribution are never independent. This also holds for $r > 2$, which can be proved by induction from the observation that mutual independence entails pairwise independence.

Assume that (Y_1, Y_2) has a negative multinomial distribution and that it is not degenerate (all the parameters are strictly positive). Let us fix $y_2 = 0$. The terms $P(y_1 = k, y_2 = 0)$ are proportional to $\Gamma(\alpha + k) p_1^k / k!$ and the terms $P(y_1 = k)P(y_2 = 0)$ are proportional to $\Gamma(\alpha + k) p_1^{*k} / k!$ where $p_1^* = p_1/(p_1 + p_0) < p_1$ so equality cannot hold for every $k \geq 0$. In conclusion, the joint distribution cannot be equal to the product of the marginal distributions.

Note that the proof above assumes $p_0 > 0$, which is a consequence of $\beta < \infty$. So as long as λ is distributed according to a proper Gamma distribution, which is a defining feature of the negative multinomial distribution, the variables cannot be independent.

From the marginal distributions we can compute the conditional distribution of $(Y_1, \ldots, Y_s)$ given $(Y_{s+1}, \ldots, Y_r)$ (and similary the distribution of any set of variables given the complentary set). Using the same approach as above, the marginal distribution is a negative multinomial that can be found to be

$$P(Y_{s+1} = y_{s+1}, \ldots, Y_r = y_r) = \frac{\Gamma(\alpha + y_{s+1} + \ldots + y_r)}{\Gamma(\alpha) y_{s+1}! \ldots y_r!} p_0^{\alpha} p_{s+1}^{y_{s+1}} \ldots p_r^{y_r} \left(\frac{1}{p_0 + p_{s+1} + \ldots + p_r} \right)^{\alpha + y_{s+1} + \ldots + y_r}.$$

The conditional distribution is computed as the ratio of the full distribution and the marginal distribution.

$$P(Y_1 = y_1, \ldots, Y_s = y_s | Y_{s+1} = y_{s+1}, \ldots, Y_r = y_r) = \frac{\Gamma(\alpha + y_1 + \ldots + y_r)}{\Gamma(\alpha + y_{s+1} + \ldots + y_r) y_1! \ldots y_s!} q_0^{\alpha + y_{s+1} + \ldots + y_r} p_1^{y_1} \ldots p_i^{y_s},$$

where we define $q_0 = p_0 + p_{s+1} + \ldots + p_r = 1 - (p_1 + \ldots + p_s)$. In other words, the distribution of $(Y_1, \ldots, Y_s)$ given $(Y_{s+1}, \ldots, Y_r)$ is negative multinomial with parameters $(\alpha + y_{s+1} + \ldots + y_r, q_0, p_1, \ldots, p_s)$.

2.4 Inference about α and p_0

We now turn our attention to the distribution of the sum of observations drawn from negative multinomial distribution. More precisely, if $(y_1, \ldots, y_r)$ is a random observation from a negative multinomial distribution with parameters $(\alpha, p_0, p_1, \ldots, p_r)$, we are interested in the distribution of $y_1 + \ldots + y_r$.

Conditionally on a given value of λ, $Y_1, \ldots, Y_r$ are independent and Poisson distributed. In regard of equation (8), this means that the conditional value of the sum is Poisson with parameter $\lambda(\gamma_1 + \ldots + \gamma_r)$. The distribution of the sum is thus negative binomial with parameters (α, p_0), where the value of p_0 is as defined in (9).

This observation will be basis of the demonstration that the vector of marginal sums is a sufficient statistics for α and p_0, which means that all the inference about these two parameters can be performed with the marginal sums. Let us consider a random sample of size n drawn from a negative multinomial distribution with parameters $(\alpha, p_0, p_1, \ldots, p_r)$ and compute its distribution conditionally on the marginal sums. The observations consist of n vectors of dimension r, denoted $(y_{k,1}, \ldots, y_{k,r})$, where $1 \leq k \leq n$, and we denote the associated marginal sum y_k. The likelihood of the sample is

$$\prod_{k=1}^{n} \frac{\Gamma(\alpha + y_{k,1} + \ldots + y_{k,r})}{\Gamma(\alpha) y_{k,1}! \ldots y_{k,r}!} p_0^{\alpha} p_1^{y_{k,1}} \ldots p_r^{y_{k,r}}.$$

The likelihood of the marginal sums is

$$\prod_{k=1}^{n} \frac{\Gamma(\alpha + y_{k,1} + \ldots + y_{k,r})}{\Gamma(\alpha)(y_{k,1} + \ldots + y_{k,r})!} p_0^{\alpha} (p_1 + \ldots + p_r)^{y_{k,1} + \ldots + y_{k,r}}.$$

The conditional distribution of the observations is found by dividing these two values, which yields

$$\prod_{k=1}^{n} \frac{(y_{k,1} + \ldots + y_{k,r})!}{y_{k,1}! \ldots y_{k,r}!} p_1^{*y_{k,1}} \ldots p_r^{*y_{k,r}},$$

where $p_s^* = p_s/(p_1 + \ldots + p_r)$. This expression does not depend on either α nor p_0, which proves that the n marginal sums represent a sufficient statistic for α and p_0.

2.5 Negative multinomial parameter estimation

The multinomial negative distribution can be fitted with the maximum likelihood method. Suppose that an IID random sample of size n is available and denote the individual observations $(y_{k,1}, \ldots, y_{k,r})$, $1 \leq k \leq n$. The likelihood of the observations is

$$L = \prod_{k=1}^{n} \frac{\Gamma(\alpha + y_{k,1} + \ldots + y_{k,r})}{\Gamma(\alpha) y_{k,1}! \ldots y_{k,r}!} p_0^{\alpha} p_1^{y_{k,1}} \ldots p_r^{y_{k,r}}.$$

According to section 2.4, we can estimate α and p_0 from the marginal sums of the observed sample, which we denote $y_1, \ldots, y_n$. The likelihood of the marginal sums is

$$L = \prod_{k=1}^{n} \frac{\Gamma(\alpha + y_k}{\Gamma(\alpha) yk!} p_0^{\alpha} (1 - p_0)^{y_k}.$$

In practice it is easier to maximize the log-likelihood $\ell = \log(L)$, which is as follows

$$\ell = \sum_{k=1}^{n} \log\Gamma(\alpha + y_k) + \log\Gamma(\alpha) - \log(y_k!) + \alpha\log(p_0) + y_k\log(1-p_0). \quad (10)$$

The maximum is found by differentiating (10).

$$\frac{\partial \ell}{\partial p_0} = \sum_{k=1}^{n} \frac{\alpha}{p_0} - \frac{y_k}{1-p_0} = 0.$$

The equation above can be rearranged to express p_0 as a function of α

$$p_0 = \frac{\alpha}{\alpha + \bar{y}}, \quad (11)$$

where $\bar{y}$ is the mean of the marginal sums (*i.e.* $\bar{y} = \sum_{k=1}^{n} y_k/n$). Differentiating with respect to α, we now obtain

$$\frac{\partial \ell}{\partial \alpha} = \sum_{k=1}^{n} \psi(\alpha + y_k) - \psi(\alpha) + \log(p_0).$$

We substitute (11) in the equation above and obtain an expression that depends on α only.

$$f(\alpha) = n\Big(\log(\alpha) - \psi(\alpha) - \log(\alpha + \bar{y})\Big) + \sum_{k=1}^{n} \psi(\alpha + y_k) = 0. \quad (12)$$

The equation above is solved by the Newton-Raphson method. For this, we use the update formula $\alpha^{(t+1)} = \alpha^{(t)} - f(\alpha^{(t)})/f'(\alpha^{(t)})$, which requires to differentiate f and to choose an initial value $\alpha^{(0)}$.

$$f'(\alpha) = n\left(\frac{\bar{y}}{\alpha(\alpha + \bar{y})} - \psi'(\alpha)\right) + \sum_{k=1}^{n} \psi'(\alpha + y_k).$$

The Newton-Raphson method is fast and converges after a few cycles. The initial value is usually chosen as $\alpha^{(0)} = 1$. Once α is known, the value of p_0 is set using equation (11).

To find the values of $p_1, \ldots, p_r$, we differentiate $\log(L)$ with respect to p_s $(1 \leq s \leq r)$ and use Lagrange multipliers. It then appears that

$$p_s = \frac{p_0 \bar{y}_s}{\alpha}, \tag{13}$$

where $\bar{y}_s$ is the mean of the s-th variable, $\bar{y}_s = \sum_{k=1}^{n} y_{k,s}/n$.

2.6 Zero-Inflation

The so called zero-inflated negative binomial (ZINB)is a mixture model with a negative binomial and a constant equal to 0. This inflates the term $P(Y = 0)$, whence the name of the distribution. Zero-inflated distributions are good models for overdispered data (which can occur as a consequence of distribution mixture). By definition, the numbers are drawn from a negative binomial distribution with parameters (α, p) with probability π and from the constant equal to 0 with probability $1 - \pi$. The distribtion is thus

$$P(Y = y) = \pi \frac{\Gamma(\alpha + y)}{\Gamma(\alpha) y!} p^{\alpha} (1-p)^y + (1-\pi)\delta_0(y),$$

where $\delta_0(y) = 1$ if and only if $y = 0$. Similarly, we set $\delta_{0^r}(y_1, \ldots, y_r) = 1$ if and only if $y_1 = \ldots = y_r = 0$ and we define the zero-inflated negative multinomial (ZINM) distribution by the formula

$$P(Y_1 = y_1, \ldots, Y_r = y_r) = \pi \frac{\Gamma(\alpha + y_1 + \ldots + y_r)}{\Gamma(\alpha) y_1! \ldots y_r!} \quad p_0^{\alpha} \quad p_1^{y_1} \ldots p_r^{y_r} + \tag{14}$$
$$(1-\pi)\delta_{0^r}(y_1, \ldots, y_r).$$

2.7 ZINM parameter estimation

Mixture distributions can be fitted using the EM algorithm, which gives a tractable solution. However, the search algorithm can be trapped in local maxima, in which case it does not return the maximum likelihood estimate. For this reason, we use another method.

Suppose, as in section 2.5, that an IID random sample of size n drawn from a zero-inflated negative multinomial is available. In order to maximize (14), we introduce $z(k_1, \ldots, k_r)$, representing the number of observations such that $Y_1 = k_1, \ldots, Y_r = k_r$. For convenience, we also introduce $z_0 = z(0, \ldots, 0)$. The log-likelihood is then written as

$$\begin{aligned}\ell &= z_0 \log(\pi p_0^\alpha + 1 - \pi) + \\ &\sum_{k_1,\ldots,k_r \neq z_0} z(k_1,\ldots,k_r)\Big(\log(\pi) + \log\Gamma(\alpha + k_1 + \ldots + k_r) - \\ &\log\Gamma(\alpha) + \alpha\log(p_0) + k_1\log(p_1) + \ldots + k_r\log(p_r)\Big).\end{aligned}$$

We first differentiate ℓ with respect to π in order to obtain a substitution expression that will yield equations independent of π.

$$\begin{aligned}\frac{\partial\ell}{\partial\pi} = z_0\frac{p_0^\alpha - 1}{\theta p_0^\alpha + 1 - \pi} + (n - z_0)\frac{1}{\pi} &= 0, \text{ which leads to} \\ \frac{z_0}{\pi p_0^\alpha + 1 - \pi} &= \frac{n - z_0}{\pi(1 - p_0^\alpha)}. \qquad (15)\end{aligned}$$

We now differentiate ℓ with respect to $p_0, \ldots, p_r$, and as in section 2.5, we need to use Lagrange multipliers.

$$\begin{aligned}\frac{\partial\ell}{\partial p_0} &= z_0\frac{\pi\alpha p_0^{\alpha-1}}{\pi p_0^\alpha + 1 - \pi} + \frac{\alpha(n - z_0)}{p_0} = \mu, \\ \frac{\partial\ell}{\partial p_i} &= \frac{y_i^*}{p_i} = \mu \ (i \neq 0), \qquad (16)\end{aligned}$$

where we have defined the statistic $y_i^* = \sum_{k_1,\ldots,k_r \neq z_0} z(k_1,\ldots,k_r)k_i$. From these equations and $p_0 + \ldots + p_r = 0$ we obtain

$$\mu = \frac{y_1^* + \ldots + y_r^*}{1 - p_0}. \qquad (17)$$

Observe *en passant* that the term $y_1^* + \ldots + y_r^*$ is simply equal to the sum of all the observations. Substituting (15) and (17) in the expression of $\partial\ell/dp_0$, we obtain

$$f(p_0, \alpha) = \frac{\alpha(n - z_0)}{p_0(1 - p_0^\alpha)} - \frac{y_1^* + \ldots + y_r^*}{1 - p_0} = 0. \qquad (18)$$

We now differentiate ℓ with respect to α and substitute (15) to obtain an equation that depends on α and p_0.

$$
\begin{aligned}
g(p_0,\alpha) &= \frac{\partial \ell}{\partial \alpha} = z_0 \frac{\pi p_0^\alpha \log(p_0)}{\pi p_0^\alpha + 1 - \pi} \\
&+ \sum_{k_1,\ldots,k_r \neq z0} z(k_1,\ldots,k_r)\Big(\psi(\alpha + k_1 + \ldots + k_r) - \psi(\alpha) + \alpha \log(p_0)\Big) \\
&= \frac{(n - z_0)\log(p_0)}{1 - p_0^\alpha} - (n - z_0)\psi(\alpha) \\
&+ \sum_{k_1,\ldots,k_r \neq z_0} z(k_1,\ldots,k_r)\psi(\alpha + k_1 + \ldots + k_r) = 0.
\end{aligned}
$$

We now need to find p_0 and α such that $f(p_0,\alpha) = g(p_0,\alpha) = 0$. This is done with the Newton-Raphson method, which, in the case of several equations requires the Hessian matrix H of the system. By definition

$$
H(p_0,\alpha) = \begin{pmatrix} \partial f/\partial p_0 & \partial f/\partial \alpha \\ \partial g/\partial p_0 & \partial g/\partial \alpha \end{pmatrix}.
$$

The update formula is analogous to the case of a single equation, namely

$$
\begin{pmatrix} p_0^{(t+1)} \\ \alpha^{(t+1)} \end{pmatrix} = \begin{pmatrix} p_0^{(t)} \\ \alpha^{(t)} \end{pmatrix} - H(p_0^{(t)},\alpha^{(t)})^{-1} \begin{pmatrix} f(p_0^{(t)},\alpha^{(t)}) \\ g(p_0^{(t)},\alpha^{(t)}) \end{pmatrix}.
$$

The terms of the Hessian matrix are computed directly by differentiating f and g with respect to p_0 and α.

$$
\begin{aligned}
\frac{\partial f(p_0,\alpha)}{\partial p_0} &= -(n - z_0)\alpha \frac{1 - (\alpha + 1)p_0^\alpha}{(p_0(1 - p_0^\alpha))^2} - \frac{y_1^* + \ldots + y_r^*}{(1 - p_0)^2} \\
\frac{\partial g(p_0,\alpha)}{\partial \alpha} &= \frac{(n - z_0)(\log(p_0))^2 p_0^\alpha}{(1 - p_0^\alpha)^2} - (n - z_0)\psi'(\alpha) \\
&+ \sum_{k_1,\ldots,k_r \neq z_0} z(k_1,\ldots,k_r)\psi'(\alpha + k_1 + \ldots + k_r) \\
\frac{\partial f(p_0,\alpha)}{\partial \alpha} &= \frac{\partial g(p_0,\alpha)}{\partial p_0} = (n - z_0)\frac{1 - p_0^\alpha + \alpha p_0^\alpha \log(p_0)}{p_0(1 - p_0^\alpha)^2}.
\end{aligned}
$$

As a gradient method, the Newton-Raphson only guarantees convergence to a local maximum, the choice of the initial values is therefore important.

To maximize the chances of finding the global maximum, the procedure described above is applied to a range of initial conditions. To determine these conditions, the number of all-null observations z_0 is decreased artificially, and the resulting dataset is fitted by the procedure descrbed in 2.5 which gives a pair (p_0, α). The pairs corresponding to distinct values of z_0 are collected and used as initial conditions for the algorithm described in this section.

In this method, there are only two free parameters, making it easier to find suitable intial conditions compared to the EM algorithm, in which there are three.

3 An emission model for ChIP-seq

The readout of ChIP-seq and similar experiments is a sequence of reads mapped to genomic windows of identical size. The zero-inflated negative multinomial distribution is a good choice[1] to describe the number of reads per window for the following reasons:

1. It is a discrete random variables with values in $\mathbb{N}$.
2. It is multidimensional and can thus accomodate multiple replicates.
3. The marginal distributions are correlated.
4. Unmappable regions of the genome inflate the windows with no read.
5. Section 2 shows that it can be interpreted as a Poisson distribution where the parameter λ varies as a Gamma variable. With this interpretation, each genomic window has a different expected read number. Conditionally on that number, the read count for a given window is a Poisson variable.

We further assume that r experiments are available. For a given genomic window and a given state x_i, the probability of observing $(y_1, \ldots, y_r)$ reads in the available profiles is

$$\pi \frac{\Gamma(\alpha + y_1 + \ldots + y_r)}{\Gamma(\alpha) y_1! \ldots y_r!} p_{0,i}^{\alpha}\, p_{1,i}^{y_1} \ldots p_{r,i}^{y_r} + (1-\pi)\delta_{0^r}(y_1, \ldots, y_r).$$

[1]One of the main weaknesses of that model is that it assumes that the distribution of the parameter λ is IID for all genomic windows. This is probably not the case, as for every profile we expect that two neighboring windows have similar expected read counts.

3.1 Estimating π and α

In section 2.1, we have argued that the negative multinomial distribution can be seen as a Gamma-Poisson process, where α is the shape parameter of the underlying Gamma distribution. The interpretation in the context of ChIP-seq experiments is that genomic windows have different expected read counts because of experimental and computational artifacts. Similarly, the parameter π is the probability that a genomic window is not mappable at all and will always have read count 0. These variations are a property of the genome and the methods used for the experiment, and not of a particular replicate. In terms of the formalism presented in section 1.1, those values are independent of the of the HMM, and they can be estimated independently.

The estimation of π and α is based on the negative controls. These experiments provide the baseline variation of read count in the given genome with the given experimental setup. Section 2.7 provides a method to estimate π and α, together with the parameters $p_0, p_1, \ldots, p_c$, where c is the number of available control experiments.

If the values of π and α can be considered constant, this is not the case of $p_0, p_1, \ldots, p_c$. Indeed, there are r profiles to be modelled by a zero-inflated multinomial distribution, and the constrain $p_0 + p_1 + \ldots + p_r = 1$ imposes that the values estimated on the negative controls alone cannot remain the same when all the profiles are considered. For this reason, we refer to estimates based on the controls only as $p_0^*, p_1^*, \ldots, p_c^*$.

In section 2.3, we have shown that $p_s/p_0 = p_s^*/p_0^*$ $(1 \leq s \leq c)$, so even if the values have to be updated, their respective ratios have to be maintained. Note that those constrains also determine the value of the ratio p_s/p_u for every pair (s, u) where $s \leq c$ and $u \leq c$. So instead of storing the values $p_0^*, p_1^*, \ldots, p_c^*$, at the end of the procedure presented in section 2.7, we store the ratios $R_1 = p_1^*/p_0^*, \ldots, R_c = p_c^*/p_0^*$.

3.2 Estimating state-dependent parameters

The remaining parameters are state-dependent, which means that their value depends on the state of the HMM. For this reason, $p_{c+1}, \ldots, p_r$ have to be further indexed by the state and they are therefore referred to as $p_{c+1,i}, \ldots, p_{r,i}$, where $1 \leq i \leq m$.

This is done through the Baum-Welch algorithm described in section 1.3. The last term of equation (5) strongly depends on the emission model, which

is why the solution had to be deferred until here. To complete the cycle of the Baum-Welch algorithm, we need to maximize the expression

$$\sum_{k=0}^{n} E_{\theta^{(t)}} \left\{ \log g_{i_k}(y_k, \theta) \middle| y_0, \ldots, y_n \right\},$$

where $g_{i_k}(y_k, \theta)$ is the probability of the emission if the state of the HMM at step k is i_k, and θ is the set of parameters. In this expression, y_k is actually an r-dimensional vector $(y_{k,1}, \ldots, y_{k,r})$, where $y_{k,s}$ is the value of the s-th variable (*i.e.* the read count in window k for experiment s). This can now be replaced by the formula of the zero-inflated negative multinomial model introduced above. More specifically, if $(y_1, \ldots, y_r) \neq 0^r$, the log-likelihood of a single emission if the HMM is in state i is

$$\begin{aligned} g_i(y_1, \ldots, y_r|\theta) = \log(\pi) + \log \Gamma(\alpha + y_1 + \ldots + y_r) - \log \Gamma(\alpha) + \\ \alpha \log(p_{0,i}) + y_1 \log(p_{1,i}) + \ldots + y_r \log(p_{r,i}), \end{aligned}$$

and if $(y_1, \ldots, y_r) = 0^r$ it is

$$g_i(y_1, \ldots, y_r|\theta) = \log(\pi p_{0,i}^{\alpha} + 1 - \pi).$$

In each state i, remember that the parameters $p_1, \ldots, p_c$ satisfy the constrain $p_{s,i}/p_{0,i} = R_s$, $1 \leq s \leq c$. In the first expression above, the constant log factorial terms have been removed because they do not depend on the state, and they are therefore neutral for the estimation process. As explained above, π and α are also state-independent and they can be considered constant. The first term above can thus be simplified to

$$\alpha \log(p_{0,i}) + y_1 \log(p_{1,i}) + \ldots + y_r \log(p_{r,i}).$$

If we denote Z_0 the set of indices k such that $y_{k,1} = \ldots = y_{k,r} = 0$, the third term of (5) can finally be computed as

$$\begin{aligned} \sum_{i=1}^{m} \sum_{k \notin Z_0} \phi_{k|n}(i) \Big(\alpha \log(p_{0,i}) + y_{k,1} \log(p_{1,i}) + \ldots + y_{k,r} \log(p_{r,i}) \Big) \\ + \sum_{i=1}^{m} \sum_{k \in Z_0} \phi_{k|n}(i) \log(\pi p_{0,i}^{\alpha} + 1 - \pi) \end{aligned} \quad (19)$$

We differentiate (19) with respect to the parameters $p_{0,i}, \ldots, p_{r,i}$, which are bound by the constrains $p_{0,i} + \ldots + p_{r,i} = 1$ and $p_{s,i} = R_i p_{0,i}$ $(1 \leq s \leq c)$. Starting with $p_{0,i}$, we obtain

$$\begin{aligned}\frac{\partial \ell}{\partial p_{0,i}} &= \frac{\alpha}{p_{0,i}} A + \frac{\pi \alpha p_{0,i}^{\alpha-1}}{\pi p_{0,i}^{\alpha} + 1 - \pi} B \\ &= \mu - R_1 \lambda_1 - \ldots - R_c \lambda_c, \end{aligned} \tag{20}$$

where $A = \sum_{k \notin Z_0} \phi_{k|n}(i)$ and $B = \sum_{k \in Z_0} \phi_{k|n}(i)$. The differentiation of (19) with respect to the other variables yields the following equalities

$$\frac{\partial \ell}{\partial p_{s,i}} = \frac{1}{p_{s,i}} \sum_{k \notin Z_0} \phi_{k|n}(i) y_{k,s} = \begin{cases} \mu + \lambda_s, & \text{if } 1 \leq s \leq c \\ \mu & \text{otherwise.} \end{cases} \tag{21}$$

For convenience, we define the statistics $y_{i,s}^{*} = \sum_{k \notin Z_0} \phi_{k|n}(i) y_{k,s}$ and the constant $C = 1 + R_1 + \ldots + R_c$. Using (20) and (21), we obtain the following expression

$$\begin{aligned} C\mu &= \frac{R_1 y_{i,1}^{*}}{p_{1,i}} + \ldots + \frac{R_1 y_{i,c}^{*}}{p_{c,i}} + \frac{\alpha}{p_{0,i}} A + \frac{\pi \alpha p_{0,i}^{\alpha-1}}{\pi p_{0,i}^{\alpha} + 1 - \pi} B \\ &= \frac{y_{i,1}^{*} + \ldots + y_{i,c}^{*} + \alpha A}{p_{0,i}} + \frac{\pi \alpha p_{0,i}^{\alpha-1}}{\pi p_{0,i}^{\alpha} + 1 - \pi} B. \end{aligned} \tag{22}$$

Starting from $p_{0,i} + p_{1,i} + \ldots + p_{r,i} = 1$ we obtain

$$p_{0,i} + \frac{1}{C\mu}(y_{i,c+1}^{*} + \ldots + y_{i,r}^{*}) = \frac{1}{C},$$

where $C\mu$ is as shown in equation (22). In summary, maximizing (19) boils down to solving the following equation

$$f(p_{0,i}) = p_{0,i} + E \left(\frac{D + \alpha A}{p_{0,i}} + \frac{\pi \alpha p_{0,i}^{\alpha-1}}{\pi p_{0,i}^{\alpha} + 1 - \pi} B \right)^{-1} - \frac{1}{C} = 0,$$

where $D = y_{i,1}^{*} + \ldots + y_{i,c}^{*}$ and $E = y_{i,c+1}^{*} + \ldots + y_{i,r}^{*}$. However tedious to derive, this expression is a function of $p_{0,i}$ only and it can thus be solved

numberically by the Newton-Raphson method. We need to compute f' and use the update formula $p_{0,i}^{(t+1)} = p_{0,i}^{(t)} - f(p_{0,i}^{(t)})/f'(p_{0,i}^{(t)})$.

$$\begin{aligned} f'(p_{0,i}) = 1 - E &\left(\frac{D + \alpha A}{p_{0,i}} + \frac{\pi \alpha p_{0,i}^{\alpha-1}}{\pi p_{0,i}^{\alpha} + 1 - \pi} B \right)^{-2} \\ &\times \left(\frac{(1-\pi)\pi\alpha(\alpha-1)p^{\alpha-2} - \pi^2 \alpha p^{2\alpha-2}}{(\pi p_{0,i}^{\alpha} + 1 - \pi)^2} B - \frac{D + \alpha A}{p_{0,i}^2} \right). \end{aligned}$$

Once the value of $p_{0,i}$ has been computed, the $p_{s,i}$ can be computed via $p_{s,i} = R_i p_{0,i}$ for $1 \leq s \leq c$ and $p_{s,i} = y_{i,s}^*/\mu$ for $c + 1 \leq s \leq r$, where μ is available from (22). The marks the end of the Baum-Welch algorithm and allows to perform another cycle.

4 Negative binomial mixture model

In a negative binomial mixture model, every observation is drawn from a finite set of negative binomial distributions. Here we will only consider the case of two distributions. More specifically we will consider that the observations are drawn from a negative binomial with parameters (α, p) with probability θ and from a negative binomial with parameters (α, q) with probability $1-\theta$. The distribution is thus

$$P(Y = y) = \frac{\Gamma(\alpha + y)}{\Gamma(\alpha) y!} \left(\theta p^{\alpha}(1-p)^y + (1-\theta) q^{\alpha} (1-q)^y \right). \tag{23}$$

Mixture distributions are commonly fitted by the EM algorithm. We suppose that an unobserved variable Z takes value 1 with probability θ and value 0 with probability $1-\theta$. Z indicates which of the two distributions the observation is drawn from. The full likelihood is

$$P(y, z) = \frac{\Gamma(a + y)}{\Gamma(\alpha) y!} \left(\theta p^{\alpha}(1-p)^y \delta_1(z) + (1-\theta) q^{\alpha}(1-q)^y \delta_0(z) \right),$$

where $\delta_0(z) = 1$ if and only if $z = 0$, and $\delta_1(z) = 1$ if and only if $z = 1$. This leads to the observation that

$$P(Z = 1 | Y = y) = \frac{\theta p^{\alpha}(1-p)^y}{\theta p^{\alpha}(1-p)^y + (1-\theta) q^{\alpha}(1-q)^y}. \tag{24}$$

The E-step of the algorithm is to write the expected log-likelihood of the distribution with respect to the conditional distribution of Z. If we write $\theta_k = P(Z_k = 1|Y = y_k)$ and drop the constant term, this quantity is

$$\ell = -n \log \Gamma(\alpha) + \sum_{k=1}^{n} \log \Gamma(\alpha + y_k) + \theta_k(\log(\theta) + \alpha \log(p) + y_k \log(1-p)) + \\ (1-\theta_k)(\log(1-\theta) + \alpha \log(q) + y_k \log(1-q)). \quad (25)$$

The M-step is to maximize (25) with respect to α, p and θ, which is done by differentiation. Introducing $\bar{y}_1 = \sum_{k=1}^{n} \theta_k y_k / \sum_{k=1}^{n} \theta_k$ and $\bar{y}_0 = \sum_{k=1}^{n}(1-\theta_k) y_k / \sum_{k=1}^{n}(1-\theta_k)$, it is easily verified that at the optimum

$$\theta = \frac{1}{n} \sum_{k=1}^{n} \theta_k$$
$$p = \frac{\alpha}{\alpha + \bar{y}_1}$$
$$q = \frac{\alpha}{\alpha + \bar{y}_0}.$$

By substituting those values in $\partial\ell/\partial\alpha$, we obtain an expression $f(\alpha)$ that depends on α only

$$f(\alpha) = n\left((\log(\alpha) - \psi(\alpha)) + \sum_{k=1}^{n} \psi(\alpha + y_k) + \theta_k \log(\alpha + \bar{y}_1) + (1-\theta_k) \log(\alpha + \bar{y}_0)\right)$$

We need to find the solution of $f(\alpha) = 0$, which is done by the Newton-Raphson method. For this, we use the update formula $\alpha_{i+1} = \alpha_i - f(\alpha_i)/f'(\alpha_i)$, where

$$f'(\alpha) = n\left(1/\alpha - \psi'(\alpha)\right) + \sum_{k=1}^{n} \psi'(\alpha + y_k) + \frac{\theta_k}{\alpha + \bar{y}_1} + \frac{1-\theta_k}{\alpha + \bar{y}_0}.$$

Summary of the jahmm EM algorithm:

Assuming that the initial parameter values $\alpha_0, \theta_0, p_0, q_0$ are available, do the following:

1. For $k = 1, \dots, n$ compute

$$\theta_k = \frac{\theta_t p_t^{\alpha_t}(1-p_t)^{y_k}}{\theta_t p_t^{\alpha_t}(1-p_t)^{y_k} + (1-\theta_t) q_t^{\alpha_t}(1-q_t)^{y_k}}.$$

2. Compute

$$\bar{y}_1 = \frac{\sum_{k=1}^n \theta_k y_k}{\sum_{k=1}^n \theta_k},$$
$$\bar{y}_0 = \frac{\sum_{k=1}^n (1-\theta_k) y_k}{\sum_{k=1}^n (1-\theta_k)}.$$

3. Update α by the Newton-Raphson scheme. Starting with $\tilde{\alpha}_0 = \alpha_t$, update the value of $\tilde{\alpha}$ with the formula $\tilde{\alpha}_{i+1} = \tilde{\alpha}_i - f(\tilde{\alpha}_i)/f'(\tilde{\alpha}_i)$, where

$$f(\tilde{\alpha}_i) = n\left((\log(\tilde{\alpha}_i) - \psi(\tilde{\alpha}_i)\right) + \sum_{k=1}^n \psi(\tilde{\alpha}_i + y_k) + \theta_k \log(\tilde{\alpha}_i + \bar{y}_1) + (1-\theta_k)\log(\tilde{\alpha}_i + \bar{y}_0), \text{ and}$$

$$f'(\tilde{\alpha}_i) = n\left(1/\tilde{\alpha}_i - \psi'(\tilde{\alpha}_i)\right) + \sum_{k=1}^n \psi'(\tilde{\alpha}_i + y_k) + \frac{\theta_k}{\tilde{\alpha}_i + \bar{y}_1} + \frac{1-\theta_k}{\tilde{\alpha}_i + \bar{y}_0}.$$

Stop iterations when $|\tilde{\alpha}_{i+1} - \tilde{\alpha}_i| < \varepsilon$ for a chosen ε, and set $\alpha_{t+1} = \tilde{\alpha}_{i+1}$.

4. Update θ, p and q by

$$\theta_{t+1} = \frac{1}{n}\sum_{k=1}^n \theta_k$$
$$p_{t+1} = \frac{\alpha_{t+1}}{\alpha_{t+1} + \bar{y}_1}$$
$$q_{t+1} = \frac{\alpha_{t+1}}{\alpha_{t+1} + \bar{y}_0}.$$

5. If the values of α, θ, p and q are stable stop the algorithm, otherwise start another cycle.

5 Zero-Inflated Negative Multinomial

The Zero-Inflated Negative Binomial (ZINB) is a mixture model with a negative binomial and a constant equal to 0. This will inflate the term $P(Y = 0)$, whence the name of the distribution. By definition, the numbers are drawn from a negative binomial distribution with parameters (α, p) with probability θ and from the constant equal to 0 with probability $1 - \theta$. The distribtion is thus

$$P(Y = y) = \frac{\Gamma(\alpha + y)}{\Gamma(\alpha)y!}\theta p^{\alpha}(1-p)^{y} + (1-\theta)\delta_0(y).$$

5.1 EM algorithm

Using the same strategy as above, we introduce a variable Z, which is equal to 1 if the numbers are drawn from the first distribution and 0 otherwise. The full likelihood distribution is

$$P(y, z) = \frac{\Gamma(\alpha + y)}{\Gamma(\alpha)y!}\theta p^{\alpha}(1-p)^{y}\delta_1(z) + (1-\theta)\delta_0(y)\delta_0(z).$$

From this, we obtain

$$P(Z = 1|Y = y) = \frac{\frac{\Gamma(\alpha+y)}{\Gamma(\alpha)y!}\theta p^{\alpha}(1-p)^{y}}{\frac{\Gamma(\alpha+y)}{\Gamma(\alpha)y!}\theta p^{\alpha}(1-p)^{y} + (1-\theta)\delta_0(y)}.$$

In reality, we can substantially simplify this formula because it takes only two distinct values.

$$P(Z = 1|Y = y) = \begin{cases} w = \frac{\theta p^{\alpha}}{\theta p^{\alpha} + 1 - \theta} & \text{if } y = 0 \\ 1 & \text{otherwise.} \end{cases}$$

To compute the expected value of the full log-likelihood with respect to the conditional distribution of Z, we label the observations such that $y_1, \ldots, y_n$ are all positive, and that the remaining n_0 observations are 0.

$$\begin{aligned} \ell = & - n \log \Gamma(\alpha) + n \log(\theta) + n\alpha \log(p) + \sum_{k=1}^{n} \log \Gamma(\alpha + y_k) + y_k \log(1-p) \\ & + n_0 \left(w \log(\theta p^{\alpha}) + (1-w) \log(1-\theta) \right). \end{aligned}$$

Differentiating with respect to θ and p, we obtain

$$\begin{aligned} \theta &= \frac{n + n_0 w}{n + n_0} \\ p &= \frac{\alpha}{\alpha + y^*}, \end{aligned}$$

where $y^* = \sum_{k=1}^{n} y_k/(n + n_0 w)$. Differentiating with respect to α and using the second equality above, we obtain the following equation, which is solved numerically with the method of Newton-Raphson.

$$f(\alpha) = -n\psi(\alpha) + (n + n_0 w)\log\left(\frac{\alpha}{y^* + \alpha}\right) + \sum_{k=1}^{n} \psi(\alpha + y_k) \tag{26}$$

$$f'(\alpha) = -n\psi'(\alpha) + (n + n_0 w)\frac{y^*}{\alpha(y^* + \alpha)} + \sum_{k=1}^{n} \psi'(\alpha + y_k) \tag{27}$$

6 Negative multinomial mixture emissions

The parameters α and θ can be estimated from the reads counts of the negative control $(y_1, \ldots, y_n)$ by the EM algorithm as shown in section 4. We now turn to the Baum-Welch algorithm under the assumptions that the observations in each profile are drawn from a negative binomial mixture distribution of which the parameters α and θ are the same.

Dropping the constants terms (also including α which is now fixed), expression (**??**) is replaced by

$$\ell = \sum_{i=1}^{m} \sum_{k=1}^{n} \phi_{k|n}(i) \log\left(\theta p_{0,i}^{\alpha}\, p_{1,i}^{z_{k,1}} \ldots p_{r,i}^{z_{k,r}} + (1-\theta) q_{0,i}^{\alpha}\, q_{1,i}^{z_{k,1}} \ldots q_{r,i}^{z_{k,r}}\right).$$

For simplicity, we introduce the terms $\theta_k(i)$ for $k = 1, \ldots, n$ and $i = 1, \ldots, m$ defined by

$$\theta_k(i) = \frac{\theta p_{0,i}^{\alpha}\, p_{1,i}^{z_{k,1}} \ldots p_{r,i}^{z_{k,r}}}{\theta p_{0,i}^{\alpha}\, p_{1,i}^{z_{k,1}} \ldots p_{r,i}^{z_{k,r}} + (1-\theta) q_{0,i}^{\alpha}\, q_{1,i}^{z_{k,1}} \ldots q_{r,i}^{z_{k,r}}}, \tag{28}$$

and the terms $\bar{z}^*_{l,i}$ for $l = 1, \ldots, r$ and $i = 1, \ldots, m$ defined by

$$\bar{z}^*_{l,i|1} = \frac{\sum_{k=1}^n \phi_{k|n}(i)\theta_k(i) z_{l,i}}{\sum_{k=1}^n \phi_{k|n}(i)\theta_k(i)},$$
$$\bar{z}^*_{l,i|0} = \frac{\sum_{k=1}^n \phi_{k|n}(i)(1-\theta_k(i)) z_{l,i}}{\sum_{k=1}^n \phi_{k|n}(i)(1-\theta_k(i))}.$$

Using a similar strategy as the EM, we can fix the $\theta_k(i)$ and treat them as constants. The solution is subject to the constaints $p_{0,i} + \ldots + p_{r,i} = 1$, $q_{0,i} + \ldots + q_{r,i} = 1$, $p_{0,i}/p_{1,i} = C_1$ and $q_{0,i}/q_{1,i} = C_2$. Using Lagrange multipliers, we easily find that

$$p_{0,i} = \frac{C_1}{C_1+1} \cdot \frac{\alpha + \bar{z}^*_{1,i|1}}{\alpha + \bar{z}^*_{1,i|1} + \ldots + \bar{z}^*_{r,i|1}},$$
$$p_{1,i} = \frac{1}{C_1+1} \cdot \frac{\alpha + \bar{z}^*_{1,i|1}}{\alpha + \bar{z}^*_{1,i|1} + \ldots + \bar{z}^*_{r,i|1}},$$
$$p_{l,i} = \frac{\bar{z}^*_{l,i|1}}{\alpha + \bar{z}^*_{1,i|1} + \ldots + \bar{z}^*_{r,i|1}}, \ (l = 2, \ldots, r).$$

and

$$q_{0,i} = \frac{C_2}{C_2+1} \cdot \frac{\alpha + \bar{z}^*_{1,i|0}}{\alpha + \bar{z}^*_{1,i|0} + \ldots + \bar{z}^*_{r,i|0}},$$
$$q_{1,i} = \frac{1}{C_2+1} \cdot \frac{\alpha + \bar{z}^*_{1,i|0}}{\alpha + \bar{z}^*_{1,i|0} + \ldots + \bar{z}^*_{r,i|0}},$$
$$q_{l,i} = \frac{\bar{z}^*_{l,i|0}}{\alpha + \bar{z}^*_{1,i|0} + \ldots + \bar{z}^*_{r,i|0}}, \ (l = 2, \ldots, r).$$

The new values of $\theta_k(i)$ are then recomputed by formula (28). Those EM-like cycles are repeated until convergence.
